# Supplementary material for: Suicidal ideation and associated factors among pregnant women attending antenatal care in Jimma medical center, Ethiopia
Source: PLoS One. 2021 Aug 25;16(8):e0255746. doi: 10.1371/journal.pone.0255746 (PMC8386870; doi:10.1371/journal.pone.0255746)
Supplement: Attachment — Submitted filename: Response to Reviewers.docx [file pone.0255746.s002.docx]

**Point by point responses for reviewers comments**

Dear Russell Kabir, Ph.D., Academic Editor, PLOS ONE

Thank you very much for giving us the opportunity to revise our manuscript “Suicidal ideation and associated factors among pregnant women attending antenatal care in Jimma, Ethiopia**”**.

We would also like to thank the reviewers for their detailed reviews and for providing us with helpful suggestions that will strengthen our manuscript and knowledge. We have gone through the comments and tried to include the responses to all the comments and suggestions.

**Comments by editor and reviewers**

**JOURNAL REQUIREMENTS:**

**Addressed accordingly.**

NB: The words or phrases that leveled as red manuscript tract changes removed in the manuscript but the yellow one are those taken managements

Point by point response to journal requirements and reviewers

A. Journal requirements
1. Please ensure that your manuscript meets PLOS ONE's style requirements, including those for file naming. The PLOS ONE style templates can be found at

Answer: we try to put the manuscript to meet PLOS ONE's style requirements

2. We suggest you thoroughly copy edit your manuscript for language usage, spelling, and grammar. If you do not know anyone who can help you do this, you may wish to consider employing a professional scientific editing service

Answer: name of the professional that edits our manuscript for language usage, spelling, and grammar service that provided: Mr. Demise Arega (BA, MA in TEEFL) from Wollo University at Department of language and literature

Email: [demisarega13@gmail.com](mailto:demisarega13@gmail.com)

3. Regarding funding- the comments provided has been accepted and modified accordingly from a-d

-As per comment the statement on the manuscript which said ” No financial support received for the study” changed to “the funders had no role in study design, data collection and analysis, decision to publish, or preparation of the manuscript”

a) Comment: Please clarify the sources of funding (financial or material support) for your study and list the grants or organizations that supported your study, including funding received from your institution

**Answer**: we received only financial support and we received only financial support from our institution Jimma university

b) Comment: State what role the funders took in the study. If the funders had no role in your study, please state: “The funders had no role in study design, data collection, and analysis, decision to publish, or preparation of the manuscript.”

**Answer:** The funders had no role in study design, data collection, and analysis, decision to publish, or preparation of the manuscript.”

c) Comment: If any authors received a salary from any of your funders, please state which authors and which funders

**Answer:** Corresponding Author

4. ORCiD –orcid id <https://orcid.org/0000-0001-8149-2534(tamrat> anbesaw)
5. Please amend your list of authors on the manuscript to ensure that each author is linked to an affiliation. Authors’ affiliations should reflect the institution where the work was done (if authors moved subsequently, you can also list the new affiliation stating “current affiliation:….” as necessary).

**Answer**: Corrected

6. Your ethics statement should only appear in the Methods section of your manuscript. If your ethics statement is written in any section besides the Methods, please move it to the Methods section and delete it from any other section. Please ensure that your ethics statement is included in your manuscript, as the ethics statement entered into the online submission form will not be published alongside your manuscript

**Answer:** comment accepted and amended

7. Please ensure that you refer to Figure 1 in your text as, if accepted

**Answer:** Figure 1 incorporated manuscript

8. We note you have included a table to which you do not refer in the text of your manuscript. Please ensure that you refer to Table 4 in your text; if accepted, production will need this reference to link the reader to the Table.

**Answer:** amended as per comment

**Comments by reviewers**

**Reviewer #1:** Important investigation. Thanks. I have some suggestions to improve the paper.

1. Comment on Title: Could be more precise. To me some unnecessary things are there.

**Author response:** Thank you. We have addressed this comment accordingly. We have revised the title as “Suicidal ideation and associated factors among pregnant women attending antenatal care in Jimma, Ethiopia” (See title, page 1 line 1-3).

2. Comment: Abstract_ Method: Please mention the name of all the instruments used.

**Response:** We have mentioned the name of the instrument as suggested. **Here is the sentence including the instrument:** EPDS, Abuse Assessment Scale (AAS), DASS-21, PSS and Maternity Social Support Scale (MSSS), and Pittsburgh Sleep Quality Index (PSQI)). (See the abstract section in the method part page 3 line 5-6).

3. Comment on Conclusion: Seems repetition of the results

Response: We addressed this comment accordingly.
4. Comment on Introduction: Suicidal ideation (SI) is a passive thought... this is not correct. It can be both active and passive. Please check the context of the reference and reconfirm

Author reply: We revised this accordingly.
5. Comment on Rationale is grossly absent. The study was done in Ethiopia not in all LAMICS. I was wondering why it has been done in Ethiopia with substantial contextualization.

**Response:** We have included the rationale of the study now. We included the following statement as the rationale of the study**:** (See introduction section page 5 paragraph 6, line 1-4).

6. Comment on Methods: August 01-30/ 2020 at Jimma,,, August 2020 is enough

**Answer:** Thank you. Amended as suggested.
7. BSc psychiatry professionals?? could you please explain.

**Answer:** those are data collectors who were participated in the study ─ Bachelor's degree holders in psychiatry.
8. Comment on Results: Table 3: comorbid depression... Please make it uniform i.e. if depression is comorbid why not anxiety and other disorder? I think mentioning depression indicate that it is comorbid

**Answer:** Thank you, Removed.
9. Discussion: I was looking for the limitations section while it should be mentioned that all instruments are not culturally validated.

**Answer: Thank you.** We have included the following statement as a limitation (See discussion section page 24 line 18-19).
10. Comment on Conclusion: Seems repetition of results.

**Answer:** Modification had been carried out.

**Reviewer #2**: a. The authors investigated an important area “Suicidal behaviour among pregnant women”. It is an area that is poorly studied. The present study found a significantly higher prevalence of suicidal ideation among Ethiopian pregnant women. This crucial information will be useful for the policy makers to augment the existing suicide prevention program.

**Author reply:** Thank you for your great appreciation; it gives us the strength to do more.
b. How the authors have selected the catchment area for the selection of participants?

**Answer:** The ANC clinic provided services for a total of approximately 9850 women every year. 9850÷12 = 820, In august (in the previous year 2018/19) was 875 mothers were attending ANC follow up, then we calculate the average number (820+875)/2 =1696÷ 2 =**848**. Finally, we had got 848, then it divided for sample size(423) I became approximately **2**. A systematic sampling technique was used to recruit participants. Based on my study the number of the 1^st^ women included in the sample was determined by the lottery method. Thus refers the 2^nd^ mother was included in the study. While the mother was found ineligible based on inclusion criteria and exclusion criteria, the next mother card number was used as a code to avoid repeated interviews of a single mother.

c. Whether the authors have used any screening tool for the assessment of psychiatric disorders? Psychiatric disorder is an important attributing factor for suicidal behavior. It is important to look for it.

**Answer:** Thank you. We have used self-reported psychiatric problems.
d. Refer to page 16: AT many places it has been mentioned (Error! Reference source not found.). Kindly provide the reference.

**Answer:** The suggestion has been completed accordingly.

**Reviewer #3:** The author appears to assess the “prevalence and risk factor of suicidal ideation among pregnant women attending antenatal care at a medical center in Southwest Ethiopia” but the title of this manuscript says “SUICIDAL IDEATION AND ASSOCIATED FACTORS AMONG PREGNANT WOMEN ATTENDING ANTENATAL CARE AT JIMMA MEDICAL CENTER, JIMMA, SOUTHWEST ETHIOPIA, 2020”. I suggest this title needs to be modified to this: “Prevalence and risk factors of suicidal ideation in Ethiopia: A case study of pregnant women attending antenatal care at Jimma medical center in Southwest Ethiopia”.

Please find below my review details:

Abstract:
− Under background, the author will need to recast the aim of the study as the year is not necessary.

**Answer:** Thank you. We revise this accordingly. Here is the sentence “suicidal ideation and associated factors among pregnant women attending antenatal care in Jimma, Ethiopia.”. (See abstract section page 3 line 5).
− Line 1 in methods, the author conducted the study with 415 pregnant women. This is not the same number in the methodology section (423 mentioned). Also, the year of the study is not mentioned at all here and this is where it is really necessary

**Answer**: Typing error amended
− Line 1 in the result, the author mentioned “current pregnancy”, he may need to recast the entire sentence.

**Answer:** Revised as: Among women who are on antenatal care was found to be ….. (See abstract section in result page 3 line 1).

Background:
− Paragraph 4 lines 2, the author made some tautologies, like “including such as psychiatric disorders”. This needs to be looked into and corrected.

**Answer:** Such as psychiatric disorders omitted from the document
− Author needs to recast the whole of Paragraph 5. The message that is being communicated is not clear.

**Answer:** The comment provided has been accepted and the long sentence has been fragmented and rearranged. Here is the revised statement “There are several risk factors for maternal suicidal behavior during the perinatal period. Some of the risk factors that exacerbate suicidal ideation women who are being unmarried, lack of support, comorbid mental illnesses, have low educational attainment, unemployment, unplanned pregnancy, history of childhood abuse, intimate partner violence, and preexisting vulnerability such as, a family history of suicide, impulsivity, and previous and/or current psychiatric diagnoses including depression.”(See introduction section page 4/5 ,paragraph 5).

− In paragraph 6, the author mentioned that there “there are no studies in Ethiopia on the subject”. I conducted a quick search and found Amare et al., 2018; Bifftu et al., 2019 and Leul et al., 2021. With this, it shows clearly that the author has not done enough literature search and needs to do more.

**Answer:** the study has been conducted in 2020, but the literature you mention is published in 2021. That mean it has one year gap. So as far as my knowledge no studies were being conducted in Ethiopia.

Methods and Materials:
− Understudy area, design and period, the author did not cite any reference.

**Answer:** Thank you. We now incorporated reference (reference number is 19).
− Author also touched on the study area only, no information was provided on the study design and period, or should one say that this entire section had not been well itemized. The author did not make clear if Source population, Study population, Inclusion and exclusion criteria, and sampling procedure techniques are all under design and period as they are just listed here.

Answer: Accepted and all are listed here in the main manuscript.

− The sample size here is 423 as against what is in the abstract.

**Answer:** It is typing error. Revised now.
− Author made a statement “similar to the previous study” in line 8 paragraph 2 under Data collection and method tools. I thought the author already said this is the first study?

**Answer:** Yes this is the first study, but to assess one of the explanatory variable (depression), the Edinburgh Postnatal Depression Scale (EPDS) was used and it was validated in different countries. So we used a similar cut score of = >13 EPND for assessing depression.

− The author may need to recast the entire Data collection procedure section. It may not be necessary to include the degree of the psychiatric professionals and stating that the supervisor is a 1st-year postgraduate student may not be necessary.

**Answer: Thank you.** Revised accordingly.

Results:
In this section, author needs to do a complete overhaul especially with typographical errors.
− Under obstetrics related characteristics of the participants, the author should delete “In the current study”, this is not needed.

**Answer:** Thank you! Comment accepted and ‘in the current study” omitted
− Line 3 and 4, Paragraph 2 under clinical and substance-related factors of the participants, the author gave percentages that are not correct. The percentages are supposed to be subset of 35 and not of the total. Therefore, the correct percentages should be 19 (54.2%), 13(37.1%), and against what it is in the manuscript.

**Answer:** Thank you for the critical comment. We are trying to show lifetime substance user in the figure, which was 87(20.9%), from this (alcohol=10.8%, khat=8.7% and ciggarate=1.4 % use substance in their lifetime, and among users 35(8.4%) are current substance users from a total of current user 8.4% ( alcohol=4.6%, khat 3.1% and cigarette smoker=1.4%). So we are put here as it is.
− The author should remove all the error messages also “reference source not found”. and current substance use

**Answer:** Removed.
− Result presented under the magnitude of suicidal ideation among pregnant women “where” are not well described. The author will need to touch on some of the other data provided in the table.

## Answer: the comments accepted and modified as The magnitude of suicidal ideation among pregnant women attending antenatal care at Jimma, Ethiopia, 2020(n=415) − Paragraph 1 under “Factors associated with suicidal ideation among pregnant women” needs to either be completely removed or recast. It looks more to me like materials and method than results.

Answer: Revised accordingly.

Discussion:
− This work is not well discussed. Author needs to conduct more literature search and discuss appropriately.

**Answer:** We revised and detailed the discussion now.

Conclusion:
− Author needs to recast and conduct more literature search to arrive at a logical conclusion

Answer: Revised accordingly.

Ethics approval and consent to participate:
− Author did not provide evidence of ethical approval for this study.

ANSWER: we provided and it was mentioned at the section Ethics approval and consent to participate:

Sincerely,

Tamrat Anbesaw, June/2021
